# Transcatheter Intervention for Multiple Valve Diseases in a Patient With Cardiogenic Shock

**DOI:** 10.1016/j.jaccas.2025.103917

**Published:** 2025-04-19

**Authors:** Sixiong Hu, Gaxue Jiang, Lu Zhang, Tian rui Liu, Yong ling Wa, Ming Bai, Jizhe XU

**Affiliations:** a Department of Cardiology, The First Hospital of Lanzhou University, Gansu, China; b Department of Echocardiography, The First Hospital of Lanzhou University, Gansu, China

**Keywords:** cardiogenic shock, multiple valve disease, transcatheter intervention

## Abstract

The combination of aortic regurgitation (AR) and mitral regurgitation (MR) is not uncommon in clinical practice, and varying degrees of regurgitation often result in severe hemodynamic disorders. Combined AR and MR often present a therapeutic challenge given the difficulty of attributing symptoms to 1 or both valves and the lack of reliable clinical trial data to guide clinical decision making. Transcatheter valve intervention has brought a new light to patients with high-risk valve disease. We report a patient with high-risk multiple valve disease and discuss the hemodynamic characteristics of the diagnostic process as well as therapeutic ideas.

## Visual Summary

**Figure:**
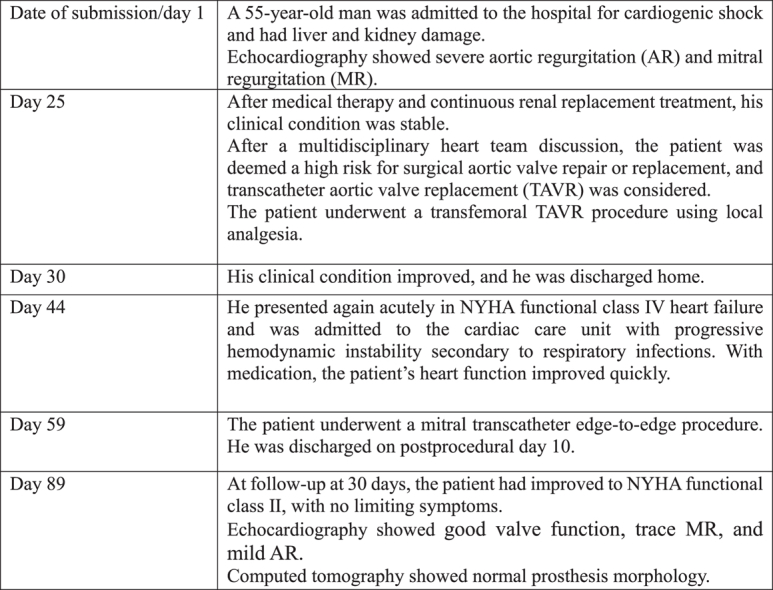

## History of Presentation

A 55-year-old male patient was admitted to the hospital for dyspnea lasting 1 year and worsening for 1 month. The patient first presented with symptoms of dyspnea in September 2023, with ultrasound indications of dilation of the ascending aorta combined with severe aortic regurgitation (AR), as well as mitral valve prolapse combined with severe mitral regurgitation (MR). The patient refused surgery and received medication. The patient developed severe heart failure following respiratory infection, combined with liver and kidney damage, in July 2024. On examination, his blood pressure was 92/50 mm Hg, his heart rate was 160 beats/min (atrial flutter), and he had orthopnea, cyanosis of the extremities, severe pitting edema of both lower extremities, and moist rales in both lungs. He had a heaving apex impulse, a severe diastolic murmur observed at the aortic valve area, and a systolic murmur observed at the mitral valve area.

**Take-Home Messages**

• We need to rationalize the characteristics of valvular diseases and the sequential order of disease from hemodynamic disorders.
• The fundamentals of treatment need to be given in terms of the relationship between hemodynamic correction and prognosis, as well as considerations of surgical accessibility.

## Past Medical History

The patient had a 10-year history of hypertension.

## Differential Diagnosis

The differential diagnosis included ruptured aortic sinus aneurysm, rheumatic valvular disease, and degenerative valvular disease.

## Investigations

The patient presented with cardiogenic shock and was dependent on vasoconstrictor drugs. The patient required volume management, cardiac function support, and effective infection control. We used continuous renal replacement therapy to remove excess fluid and toxins. With the combination of these measures, the patient’s condition initially improved.

**Figure 1:**
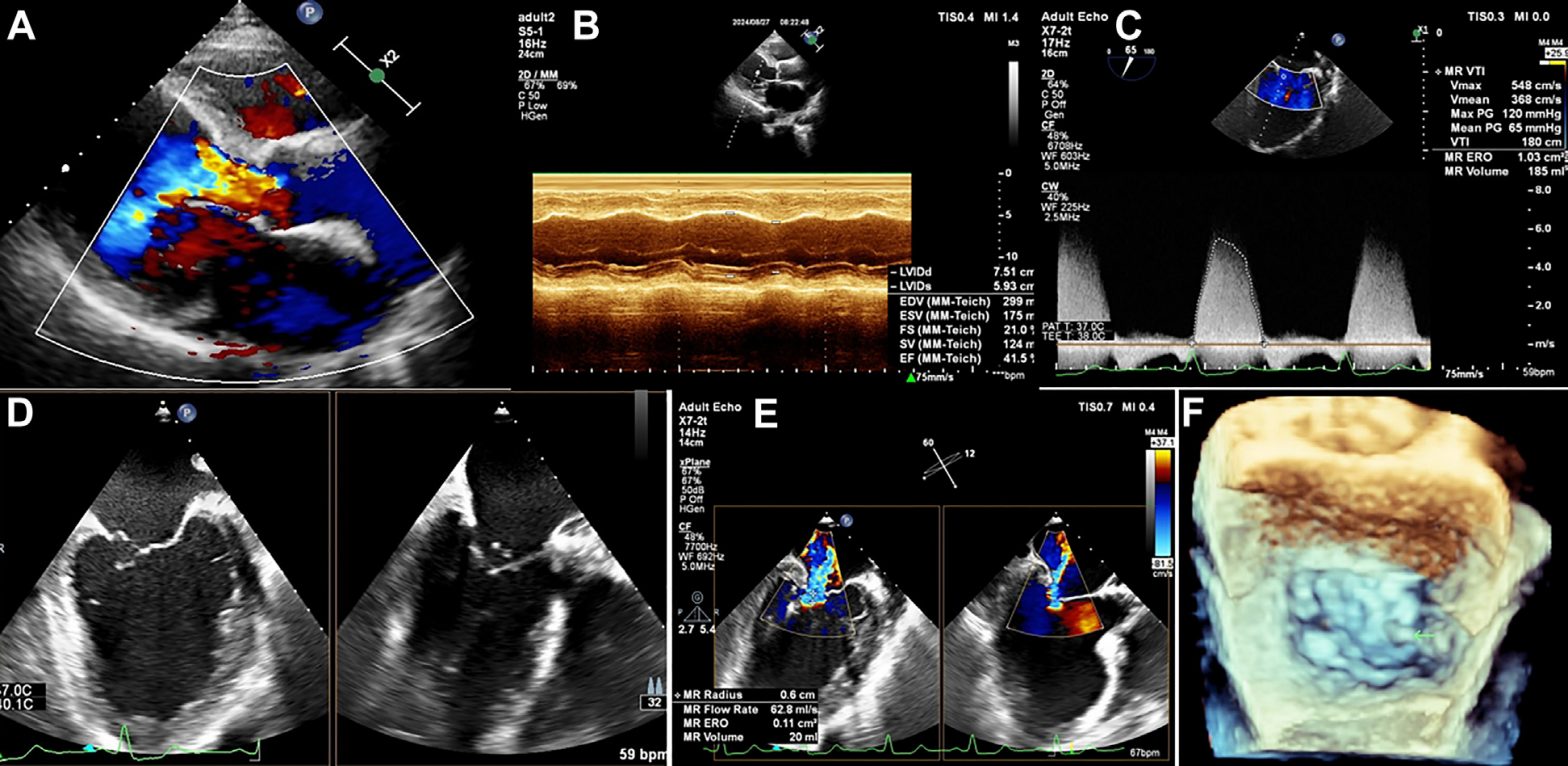
Evaluation of Aortic Regurgitation and MR by Thoracic and Transesophageal Echocardiography (A) Aortic regurgitation; vena contracta = 12 mm. (B) Left ventricular dilation; left ventricular ejection fraction = 41.5%. (C) Mitral regurgitation (MR); effective regurgitant orifice (ERO) area = 1.03 cm^2^. (D) Biplane images in bicommissural and left ventricular outflow tract views by transesophageal echocardiography. (E) Effective regurgitant orifice area and proximal isovelocity surface area radius. (F) 3-dimensional en face surgery view; the arrow shows ruptured chordae tendineae. 2D = 2-dimensional; bpm = beats/min; EDV = end-diastolic volume; EF = ejection fraction; ESV = end-systolic volume; LVIDd = left ventricular internal diameter in diastole; LVIDs = left ventricular internal diameter in systole; max = maximum; PG = pressure gradient; SV = stroke volume; V = velocity; VTI = velocity time integral.

Transthoracic echocardiography (TTE) showed moderate concentric left ventricular (LV) dilation with an LV ejection fraction (LVEF) of 41%. There was severe AR with left coronary cusp prolapse. According to the Carpentier classification of AR, type III was the main mechanism of AR in this patient.^1^ Transesophageal echocardiography (TEE) showed prolapse of the mitral valve in P3 with rupture of the tendon chords combined with a dilated mitral annulus (Figures 1A to 1F).

Surgery is preferred for multiple valve diseases according to guideline recommendations.^2^^,^^3^ The patient had a Society of Thoracic Surgeons predicted risk of mortality score of 9.5%, which is considered a high surgical risk. Guidelines suggest that in patients with surgical high-risk AR, transcatheter aortic valve replacement (TAVR) may be considered under conditions where the aortic root anatomy is suitable.^2^ MR often has complex mechanisms. AR caused LV enlargement, and chordae rupture caused degenerative MR. Therefore, the mechanisms of this MR comprised Carpentier types I, II, and IIIb. AR was improved after TAVR, the preload was reduced, and the FMR could be improved. We think maybe the mitral valve therapy strategy could be changed after TAVR and guideline-directed medical therapy (GDMT).

## Management

**Figure 2:**
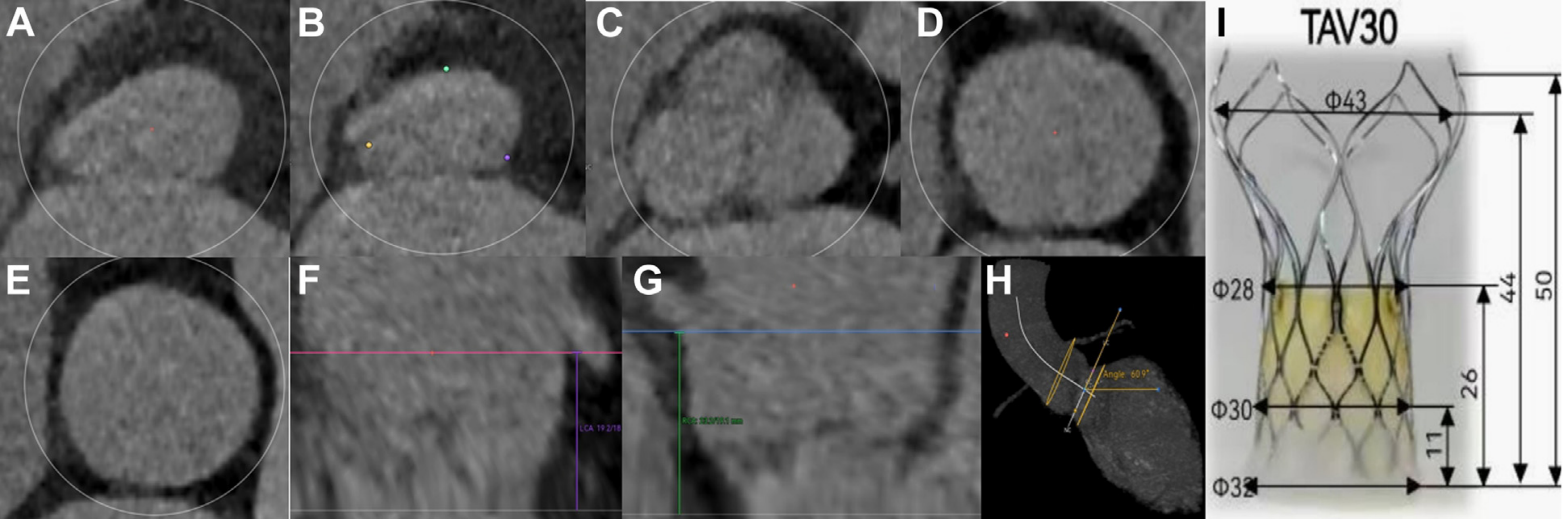
Evaluation of the Aortic Root and the Degree of Oversizing Rate by Computed Tomography Angiography (A) The left ventricular outflow tract diameter (area) was 26.7 mm; compression ratio: (323−26.7) / 32 = 16.6%. (B) The annular diameter (area) was 26.2 mm; compression ratio: (30−26.2) / 30 = 12.7%. (C) The sinus of Valsalva diameter (area) was 33.9 mm. (D) The sinotubular junction diameter (area) was 33.3 mm. (E) The ascending aorta diameter (area) was 37 mm; compression ratio: (43−37) / 43 = 14%. (F) The left coronary artery height was 19.2 mm. (G) The right coronary artery height was 19.1 mm. (H) The maximal intensity projection showed any calcification in the aortic root. (I) Radial line of the VitaFlow. TAV = transcatheter aortic valve.

**Figure 3:**
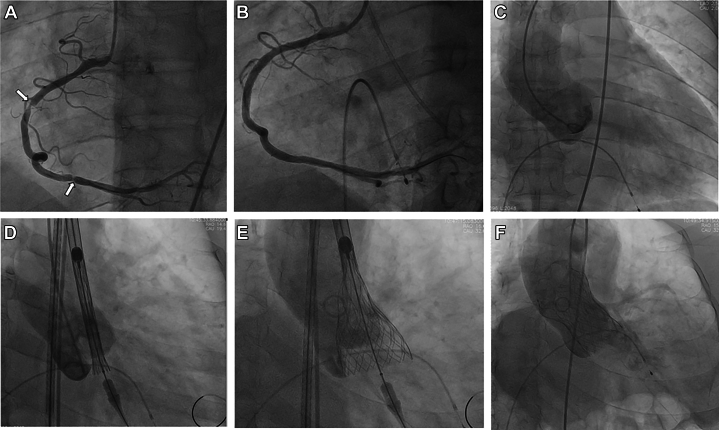
Procedure of Coronary Angiography, Percutaneous Coronary Intervention, and Transcatheter Aortic Valve Replacement Using Local Anesthesia Severe coronary local stenosis in the right coronary artery. (B) Post stents implanted in the right coronary artery. (C) Aortic root angiography by power injection. (D) Initial positioning of the valve. (E) Confirmation of valve implantation position through angiography. (F) Valve fully released and trace paravalvular leak checked by angiography.

Considering the patient’s basal status for tolerance of the procedure and anesthesia time, we chose to achieve TAVR using local anesthesia and prepared extracorporeal membrane oxygenation as a safeguard. Mitral transcatheter edge-to-edge repair (TEER) requires the use of general anesthesia, so we decide to separate the 2 procedures. Our anatomical analysis of the aortic root showed mild thickening of the valve, no calcification, and a low risk of coronary obstruction. All 3 levels can be anchored according to the diameters of the level of the LV outflow tract, the annulus, and the ascending aorta, all of which provided reliable anchoring force, and the valve leaflets were slightly thickened, which provided a positive impact on the success of transfemoral TAVR.^4^^,^^5^ We decide to use an off-label self-expanding valve and evaluate the degree of oversizing rate (Figures 2A to 2I). We first performed coronary angiography, which showed 2 severe stenoses in the right coronary artery and implanted 2 stents. The right femoral artery of the patient was chosen for TAVR as the primary vascular access with a 22-F-long introducer. A 30-mm VitaFlow self-expanding valve (MicroPort) was advanced through a 22-F sheath. Moving the pigtail catheter into the noncoronary cusp and adopting a cusp-overlapping strategy, the catheter was positioned accurately. The initial positioning of the implant should make the ring of the tip capsule about 2 mm below the annulus. Rapid pacing was performed at the rate of 160 to 180 beats/min to lower the systolic blood pressure below 60 mm Hg. Homogeneous velocity released the valve by an electric delivery system at the two-thirds of the valve overall. Evaluation by radiography and TTE focused on the valve position, anchoring, morphology, and paravalvular leakage. Rapid pacing at the rate of 140 beats/min released the valve stably. Angiography showed a correct position with mild perivalvular leakage according to the Selles grading criteria (Figures 3A to 3F, Video 1). TEE showed that the bioprosthesis was in a normal position, with an LVEF of 42%, and an aortic valve flow velocity of 1.6 m/s. Electrocardiography suggested atrial fibrillation combined with left bundle branch block. The patient was discharged from the hospital on the fourth day after TAVR.

**Figure 4:**
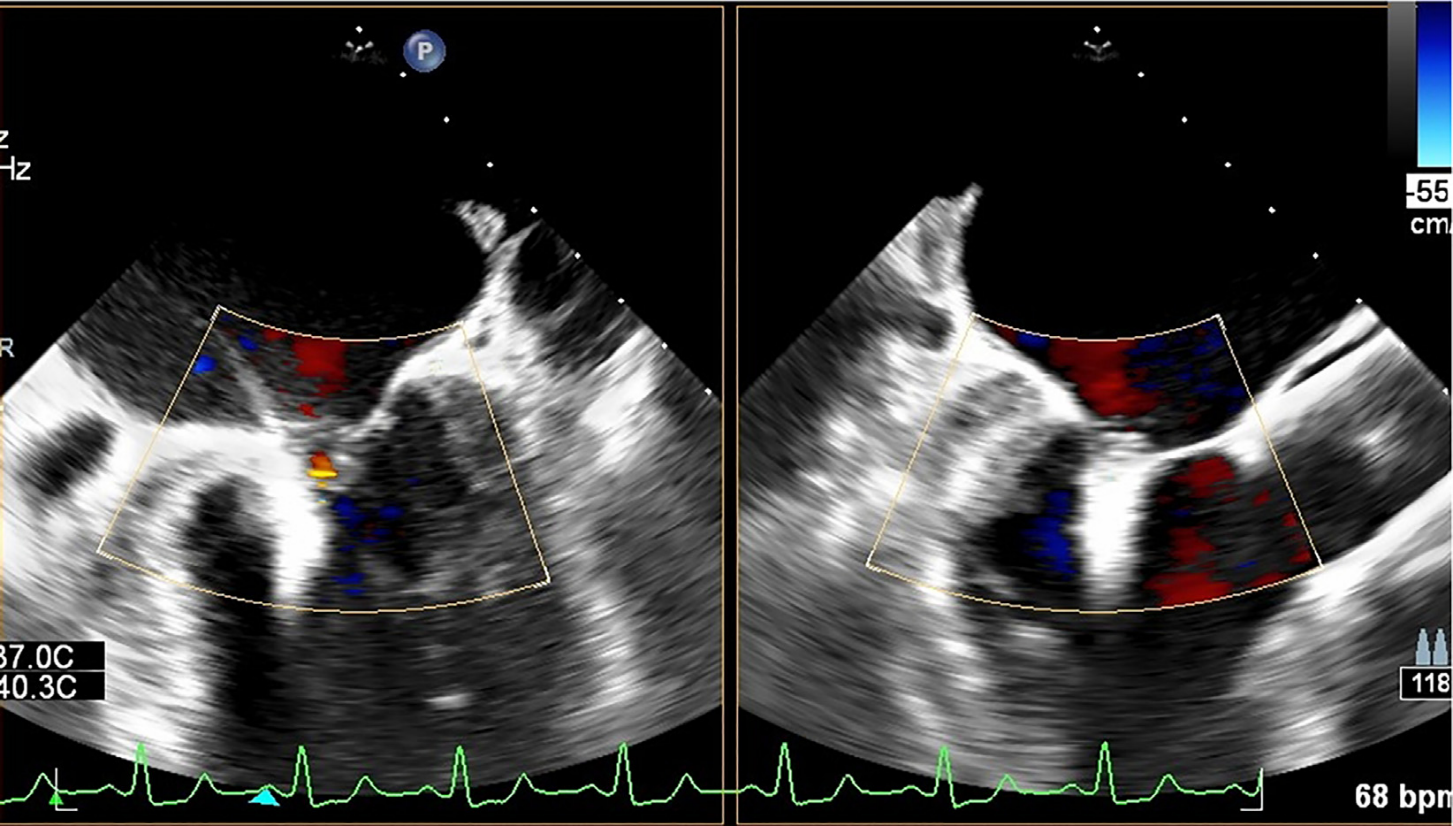
Final Result of Transcatheter Edge-to-Edge Repair by Transesophageal Echocardiography The first clip sized by 06 (width) and 12 (length) was implanted in posterior commissural leaflet P3. And another clip sized by 06 (width) and 12 (length) was implanted in A3 to P3 from shoulder to shoulder. Transesophageal echocardiography showed mitral regurgitation reduced from 4+ to trace. bpm = beats/min.

The patient’s cardiac function deteriorated again 2 weeks later as a result of a respiratory infection. The patient was in sinus rhythm, which suggested that the TAVR was effective. Subsequently, we used the DragonFly transcatheter mitral TEER system to help this patient. The first clip, 0612 (width by length), locked posterolateral commissure to A3P3, and the second clip, 0612, locked A3 to P2P3. MR was reduced from 4+ to 1+ by TEE evaluation (Figure 4, Video 2, Video 3, Video 4, Video 5), and the transvalvular pressure difference was 4 mm Hg. Table 1 is a list of equipment used for the procedures in this case.

**Table 1:** List of Equipment Used in This Case

Transcatheter aortic valve replacement
Imaging
• Transthoracic echocardiography (TTE) (GE Medical System) ○ M5Sc TTE probe
Access
• Ultrasound machine (GE Medical System)
• Micropuncture needle and wire
• 0.035-inch J wire and 7-F sheath
• 2 ProGlide (Abbott Vascular)
• Lunderquist Extra Stiff Wire Guide (Cook)
• 22-F sheath (APT Medical)
Transcatheter aortic valve replacement device
• VitaFlow transcatheter aortic valve system (MicroPort)
Mitral transcatheter edge-to-edge repair
Imaging
• Transesophageal echocardiography (TEE) (Philips Healthcare) ○ 0.035-inch X7 TEE probe
Access
• Ultrasound machine (Philips Healthcare)
• Micropuncture needle and wire
• 0.035-inch J wire and 7-F sheath
• 1 ProGlide (Abbott Vascular)
• Supra stiff guide wire (Boston Scientific)
• 18-F Cook sheath (Cook Medical)
Transseptal puncture
• Transseptal needle (Boston Scientific)
• SL-1 sheath (Abbott Vascular)
• 0.032-inch wire
• Supra stiff guide wire (Boston Scientific)
Mitral transcatheter edge-to-edge repair device
• DragonFly system (steerable guide catheter [SGC] and clip delivery system [CDS]) (Valgen Medtech)

**Visual Summary:** Timeline of Treatment

Date of submission/day 1	A 55-year-old man was admitted to the hospital for cardiogenic shock and had liver and kidney damage. Echocardiography showed severe aortic regurgitation (AR) and mitral regurgitation (MR).
Day 25	After medical therapy and continuous renal replacement treatment, his clinical condition was stable. After a multidisciplinary heart team discussion, the patient was deemed a high risk for surgical aortic valve repair or replacement, and transcatheter aortic valve replacement (TAVR) was considered. The patient underwent a transfemoral TAVR procedure using local analgesia.
Day 30	His clinical condition improved, and he was discharged home.
Day 44	He presented again acutely in NYHA functional class IV heart failure and was admitted to the cardiac care unit with progressive hemodynamic instability secondary to respiratory infections. With medication, the patient’s heart function improved quickly.
Day 59	The patient underwent a mitral transcatheter edge-to-edge procedure. He was discharged on postprocedural day 10.
Day 89	At follow-up at 30 days, the patient had improved to NYHA functional class Ⅱ, with no limiting symptoms. Echocardiography showed good valve function, trace MR, and mild AR. Computed tomography showed normal prosthesis morphology.

## Outcome and Follow-Up

**Figure 5:**
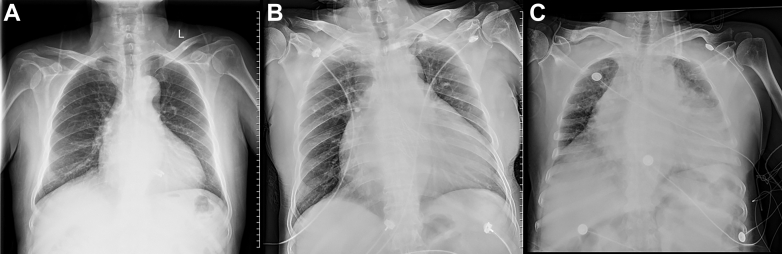
Evolution of Chest Radiography From First Admission to Recovery (A) Chest radiograph showed that the heart/chest ratio was 0.63 at discharge and recovery. (B) The heart/chest ratio was 0.81 at the second admission to the cardiac care unit. (C) The heart/chest ratio was 0.9 at the first admission to the cardiac care unit with cardiogenic shock and severe pulmonary edema.

**Figure 6:**
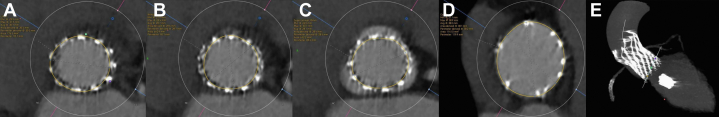
Computed Tomography Scan at 3-Month Follow-Up Morphology of the prosthesis and the relationship with surrounding tissues. (A) Left ventricular outflow tract. (B) Annulus. (C) 10 mm above the annulus. (D) Corolla. (E) The overall of the morphology.

Postoperative treatment included angiotensin receptor-neprilysin inhibitor, beta-blockers, sodium-dependent glucose transporters 2, and mineralocorticoid receptor antagonist agents. Because of stent implantation in the patient’s coronary arteries and the comorbidity of atrial fibrillation, with a CHA_2_DS_2-_VA score of 3 and a HAS-BLED score of 3, the patient was indicated for anticoagulant therapy but had a high risk of bleeding and needed to be closely monitored for bleeding. After discharge, the patient was treated with clopidogrel and dabigatran. TTE showed good valve function and trace MR and mild AR at 1 month after intervention. Chest radiography showed that the heart shadow gradually shrank, and pulmonary congestion improved (Figures 5A to 5C). Computed tomography showed normal prosthesis morphology and no leaflet thrombus at 3-month follow-up (Figures 6A to 6E). The patient’s NYHA functional classification improved from class IV to class II.

## Discussion

An observational study from China found that regurgitation accounts for the majority of valve heart diseases, and the most common dual dysfunction is aortic valve combined with mitral valve.^6^ Among these diseases, it is not uncommon to see patients with low-grade valve regurgitation, and when both valves have severe regurgitation at the same time, severe hemodynamic disorders can lead to decreased cardiac function, surgical risk becomes high, and the prognosis is poor. The diagnosis and treatment of heart valve disease have improved dramatically in recent years, but data to guide clinical decision making in patients with multiple valve disease remain limited.^3^

Transcatheter valve intervention has become an effective protocol for saving high-risk patients. TAVR in patients with AR is difficult and requires strict anatomical conditions for the valve annulus, and preoperative analysis of the aortic root anatomy is particularly important. For MR, there are often complex mechanisms, which determine the strategy of mitral TEER. A domestic study on TAVR in treating combined AR and MR found that severe AR manifests with varying degrees of functional MR (FMR), which can be improved after TAVR.^7^ The MATTERHORN (A Multicenter, Randomized, Controlled Study to Assess Mitral vAlve reconsTrucTion for advancEd Insufficiency of Functional or iscHemic ORigiN) study also confirmed that in patients with FMR, mitral TEER is not inferior to surgery.^8^ Therefore, mitral TEER is feasible for different mechanisms of MR, for which the mechanism and degree of MR will improve after downstream TAVR and GDMT treatment, thus leading to a rational surgical strategy. Patients with a high risk of combined valve disease have rapid progression and high surgical risk, and intraoperative remedial extracorporeal circulation surgery is extremely risky in the event of acute complications such as valve displacement and aortic dissection. Therefore, for this group of patients, we need to do a good job in the assessment of surgical risk, the choice of surgical methods, the timing of surgery, as well as strict follow-up.

## Conclusions

Severe AR and MR combined with acute heart failure are characterized by high surgical risk and mortality, and initial correction of the heart failure state consists of medication, reasonable imaging, and assessment of the degree of surgical tolerance. Transcatheter intervention can be performed in stepwise fashion to correct the hemodynamic disorder and improve the patient’s clinical status and prognosis.

## Funding Support and Author Disclosures

This work was supported by the Provincial Science and Technology Plan (funding number: 23YFWA0012). The authors have reported that they have no relationships relevant to the contents of this paper to disclose.

## Appendix

Video 1 The Process of Bioprosthesis Release and with Mild Perivalvular Leak

Video 2 TEE Commissural View Before TEER

Video 3 TEE X-Plane View Before TEER

Video 4 TEE LV Outflow Tract View Before TEER

Video 5 TEE 3-Dimensional View After TEER
